# Modeling Root-Knot Nematode Regulation by the Biocontrol Fungus *Pochonia chlamydosporia*

**DOI:** 10.3389/ffunb.2022.900974

**Published:** 2022-07-26

**Authors:** Aurelio Ciancio, Ileana Miranda Cabrera, Leopoldo Hidalgo-Diáz, Ana Puertas, Yoannia Castillo Duvergel

**Affiliations:** ^1^CNR, Istituto per la Protezione Sostenibile delle Piante, Bari, Italy; ^2^Centro Nacional de Sanidad Agropecuaria (CENSA), San José de las Lajas, Mayabeque, Cuba; ^3^Laboratorios Biológicos Torres S.A., San Isidro, Nicaragua; ^4^Universidad de Granma, La Habana, Cuba

**Keywords:** fungus, *Meloidogyne*, modeling, parasitism, population dynamics

## Abstract

Two models of increasing complexity were constructed to simulate the interactions between the root-knot nematode (RKN) *Meloidogyne incognita* and the biocontrol fungus *Pochonia chlamydosporia* var. *catenulata* in a rhizosphere microcosm. The models described discrete population dynamics at hourly rates over a 6-month period and were validated using real parasitism and nematode or fungus data. A first, general *Pochonia*–nematode–root model (GPNR) used five functions and 16 biological constants. The variables and constants describing the RKN life cycle included the rates of egg production, hatching, juvenile (J2), and mature female development, including root or nematode self-density-dependent factors. Other constants accounted for egg parasitism, nematode-induced root losses, growth, and mortalities. The relationship between nematodes and fungal propagules showed density dependence and cyclic variations in time, including an attractor on the propagules and J2 phases space. The simulations confirmed a *P. chlamydosporia* optimal initial density of 5 · 10^3^ propagules · cc soil^-1^, as usually applied in assays. The constants used in GPNR showed adherence to the nematode biology, with 10^3^ eggs per egg mass, a 10-day average lifespan of J2, with 2 days required to enter roots, and adult lifespan lasting 24 days. The fungus propagule lifespan was 25 days, with an average feeder root lifespan lasting around 52 days. A second, more complex *Pochonia*–nematode–root detailed model (GPNRd) was then constructed using eight functions and 23 constants. It was built as GPNR did not allow the evaluation of host prevalence. GPNRd allowed simulations of all RKN life stages and included non-parasitic and parasitic fungus population fractions. Both GPNR and GPNRd matched real J2 and fungus density data observed in a RKN biocontrol assay. Depending on the starting conditions, simulations showed stability in time, interpreted as effective host regulation. GPNRd showed a fungus cyclic relationship with the J2 numbers, with prevalence data close to those observed (38.3 *vs*. 39.4%, respectively). This model also showed a further density-independent nematode regulation mechanism based on the *P. chlamydosporia* switch from a non-parasitic to a parasitic trophic behavior. This mechanism supported the biocontrol of *M. incognita*, also sustained by a concomitant increase of the root density.

## Introduction

The use of chemicals to control plant-parasitic nematodes (PPN) is actually restricted in several countries due to concerns on pesticide environmental impact, consumers’ preference for organic food, legislation for agriculture, or simply because of market unavailability. Among available management technologies, the exploitation of one or more biocontrol agents (BCA) is investigated as a possible, sustainable alternative.

Several PPN have a severe impact on yields, mostly due to crop intensification, low levels of tolerance/resistance of attacked plants, or insufficient natural regulation exerted by the antagonistic bacteria and fungi resident in the rhizosphere microcosm (Coyne et al., 2018; Topalović and Vestergård, 2021). However, studies on nematode density changes showed that a natural regulation may occur in undisturbed soil conditions (*i*.*e*., non-agricultural soils, coastal vegetations) due to a complex of rhizosphere microorganisms (Kerry, 2001; Piskiewicz et al., 2007). Several authors also reported a PPN soil suppressivity related to soil biotic components (Olatinwo et al., 2006; Bent et al., 2008). Many PPN-antagonistic microorganisms have a strict host preference and often show high host dependence or even fastidious metabolism (Stirling, 2014). Thanks to molecular approaches, it is now clear that the PPN antagonists also possess a high level of diversity as shown by the increasing number of microbial species known for nematode parasitism reported in the last decade (Huang et al., 2005; Bishop et al., 2007; Wang et al., 2007; Rae et al., 2010). Given the huge diversity of microorganisms present in soil (Torsvik et al., 1996; Lim et al., 2010), it is likely that the number of BCA taxa will still increase in the future. In fact, many specialized spore-forming bacteria still remain undescribed due to difficulties inherent to their isolation and/or cultivation (Stirling, 2014)

In spite of diversity, the efficacy of many BCA is often not sufficient to control PPN, in particular in soils altered by anthropic activities, including highly intensive crops. Other factors affecting PPN natural regulation include pest virulence, high nematode densities due to continuous croppings and monocultures, as well as changes induced in the soil food webs by cultivation practices. Several biocontrol assays carried out at the field scale often did not confirm the results observed in greenhouse trials, showing varying levels of success often related to the specificity of the antagonists applied, the complexity of the ecosystems under examination, or the intricate relationships linking soil microbiome and PPN (Jaffee, 1992; Kerry, 2001; Lopez-Llorca et al., 2008; Topalović and Vestergård, 2021).

Root-knot nematodes (RKN, *Meloidogyne* spp.) are severe pests of food crops in many agricultural systems worldwide. In particular, *M. incognita* is one of the most important and widespread nematode pests affecting horticultural crops in Mediterranean climates and fruit or pulse crops in tropical and subtropical environments. Attempts to develop RKN biological control strategies considered a number of specialized fungi or bacteria (Stirling, 2014). The suppression of *M. incognita* has been observed in some host–parasite studies, but few information is available on the mechanisms and factors affecting the persistence and density changes of both PPN and BCA populations in time (Bailey et al., 2008).

Modeling plant pests is helpful in the investigation of several host–plant interactions and may yield useful information to be exploited in the integration of management strategies and biological control (Bailey et al., 2008). Modeling the PPN density changes in a soil/root microcosm may allow the identification of main factors active in such systems. The multiple tri-trophic interactions linking roots, PPN, and antagonists, however, originate a very complex system. It requires knowledge about the many parameters describing the nematode life stages, the interactions with the BCA present in the microcosm, as well as their capabilities for effective and durable host regulation. Descriptive variables should also account for the effects of roots and of other environmental factors at work in the tri-trophic interaction system.

Models available for application to soil fungi (Gilligan, 1985; Boswell and Hopkins, 2009) include a number of systems proposed to describe the epidemiology and management of fungal diseases (Van den Bosch and Gilligan, 2008; Castle and Gilligan, 2012). Their applications to the epidemiology of air-borne pathogens or invasive plant parasitic fungi were based on host density and susceptibility, over the areas in which the disease spreads (Gilligan and Van den Bosch, 2008). However, a few models are known for the rhizosphere microcosms in which PPN hatch from eggs located on the root and migrate only up to a few centimeters in a very small soil volume, within an environment in which nematophagous fungi or nematode-parasitic bacteria are present. In this case, the BCA attacking the nematodes are confined to a small microcosm, with propagule movements often affected by physical factors such as percolation, soil structure, and diameter of pores and particles (Jaffee, 1992).

The first models developed for PPN initially aimed at describing their life cycles and population changes. Seinhorst (1967) showed that initial nematode densities are directly related to the root damage induced and inversely linked to the nematode multiplication rates. These theoretical relationships were experimentally verified for several species, including potato cyst nematodes (Phillips et al., 1998). Other models were developed for nematodes with more than one generation per year, *i*.*e*., *Meloidogyne arenaria* (Ferris, 1976). A time-discrete system built for the sugarbeet cyst nematode, *Heterodera schachtii*, provided forecasts at yearly intervals for larval stages, eggs, and adults (Schmidt et al., 1993). Other models applied to PPN allowed the evaluation of the damage induced by two species using environmental factors and root system data (Tixier et al., 2006). All these models were useful to evaluate nematode density changes as affected by plant growth.

Modeling applications for nematode biocontrol were mainly based on a microcosm-scale analysis, in some cases describing the effects of endoparasitic fungi, such as the endoparasitic fungus *Hirsutella rhossiliensis*, or host-specific bacterial parasites (*i*.*e*., *Pasteuria* spp.). Most of these approaches considered density-dependent relationships (Jaffee, 1992; Atibalentja et al., 1998; Ehwaeti et al., 2000; Bailey et al., 2008).

Describing the regulatory effects exerted by microorganisms present in the plant–nematode system increases the system complexity, whose result may differ from a simple sum of effects. Given the large number of microbial species present in soil, modeling nematode biocontrol should be initially simplified to one or a few more antagonists. Models should realistically simulate the relationships that eventually get established in the soil microcosm after the introduction of one BCA through, *i*.*e*., inundative treatments or inoculation. Although simplified, these models may allow the identification of key variables affecting host regulation, including the insurgence of host PPN stability at non-damaging density levels or even the emergence of a nematode-suppressive effect.

The soil fungus *Pochonia chlamydosporia*, an egg parasite that showed an evolutive adaptation and a parasitic specialization towards several PPN (Kerry, 2001), was examined in this study. The fungus behaves in the rhizosphere also as a saprotroph or an endophyte, eliciting a plant defense response (Lopez-Llorca et al., 2008; Maciá-Vicente et al., 2009; Rosso et al., 2011; Pentimone et al., 2019). It also has potential as an effective biocontrol agent and rhizosphere management tool (Kerry, 2001; Maciá-Vicente et al., 2009; Manzanilla-López et al., 2013).

Different isolates of *P. chlamydosporia* have been tested worldwide for the biocontrol of *M. incognita* (Hidalgo-Díaz et al., 2000; Kerry, 2001; Verdejo-Lucas et al., 2003). Studies on the interactions of *P. chlamydosporia* and *M. incognita* in the pathozone showed that the fungus did not affect the nematode dynamics in the root space nor in the site of second-stage juvenile (J2) root penetration. The probability of root infection appeared related to the density of eggs in a small volume around roots and to the number of migrating J2 reaching the root tip. Simulations showed that the fungus acted as an egg parasite rather than providing a barrier, lowering root penetration by J2 (Bailey et al., 2008).

Models may provide benefits, including the possibility of identifying the best moment for the inoculation of a biocontrol agent, the optimal amount of its inoculum applied to maximize its biocontrol efficacy, as well as the possibility to simulate and thus investigate its behavior once introduced in soil.

The objective of this study was the construction and evaluation of reliable models describing the relationship between *P. chlamydosporia* and *M. incognita* in a root microcosm. Two models of increasing complexity, simulating the density changes of *P. chlamydosporia* and *M. incognita* in the rhizosphere of a nematode-parasitized plant, were constructed. The aim was to determine the main factors governing the dynamics of either *P. chlamydosporia* and *M. incognita* and the conditions eventually leading to a stable nematode regulation. A graphic construction of relationships among roots, fungus, and nematodes was used as starting point, as applied in general models of invertebrate species microparasites (Anderson and May, 1981). Two discrete systems of increasing complexity were then constructed, accounting for more than one nematode generation. They were tested *versus* real data to describe the effects of the fungus trophic behavior and the potential of its biocontrol activity, and their potential was also discussed.

## Materials and Methods

### Nematode and Fungus Counts

Data on the effects of *P. chlamydosporia* var. *catenulata* (isolate IMI SD187) on *M. incognita* attacking tomato were used for model validation (Puertas, 2007). The host and fungus density values were obtained from a field site located at the National Research Institute of Plant Health, La Habana. The area had been previously planted with tomato and sweet pepper which showed severe RKN infestations. The chlamydospores used as inoculum were extracted from colonized rice after 16 days of incubation at 25°C, using a MycoHarvester™, and counted using a hemocytometer. The fungus was applied by adding 5,000 chlamydospores · g^-1^ soil — to a depth of 15 cm — mixed with organic matter (cattle manure) prior to application at a rate of 1 kg organic matter · m^-2^. The plots were planted with two consecutive tomato crops (cv. Amalia). Soil cores were taken at random from each plot at the time of fungal application, then 2 months later, and at the harvest of the first crop. Similarly, cores were taken from the second crop at planting time, 2 months later, and at harvest.

The data included the colony-forming units (CFUs) obtained from soil and roots, as a colonization variable, measured on a semi-selective medium on Petri dishes in two replicates as described (Kerry et al., 1993). The means of CFUs from roots and soil were used for validation, as the models aimed at simulating a rhizosphere space volume. For egg parasitism (prevalence), data were obtained from disrupted egg masses by pouring 0.2 ml on Petri dishes with water agar and antibiotics. After 48 h at 25°C, the dishes were observed with a light microscope at ×200 to detect fungus emergence from eggs and to calculate prevalence. The J2 in 100 g of rhizosphere soil was collected through sieving funnels in 5 days and counted at ×50. For females, 3 subsamples of 1 g of roots per plant were collected, assuming a single female for smaller galls or counting their numbers in case of larger ones. Pooled data were used for validating the models using the phases space given by CFUs and simulated or observed J2 densities, assuming equivalent numbers per g and cubic centimeter of soil.

### General Model (GPNR)

**Figure 1:**
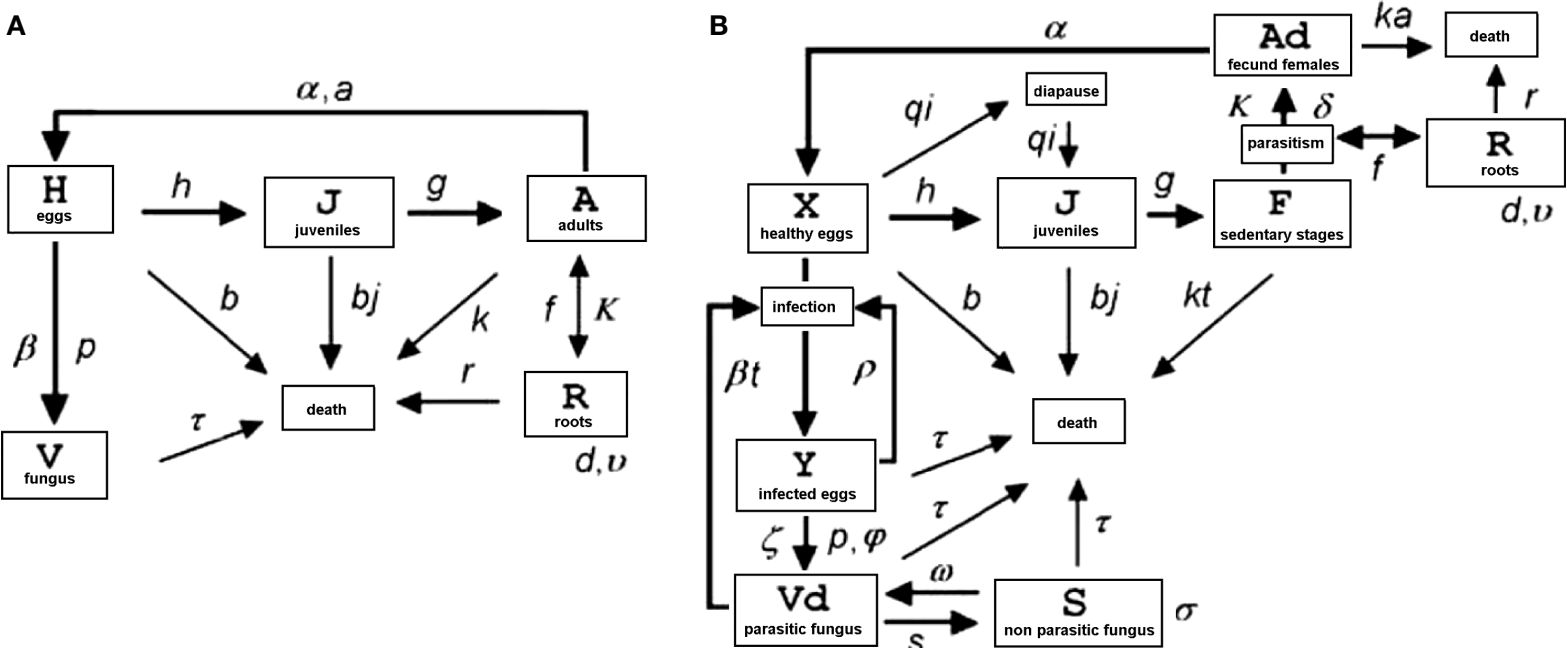
Diagrams of models GPNR **(A)** and GPNRd **(B)**. Both models describe the relationships among the root-knot nematode *Meloidogyne incognita*, the parasitic fungus *Pochonia chlamydosporia*, and the host plant root in a simulated rhizosphere microcosm (for variables, constants, and dimensions see **Table 1**).

The basic approach applied by Anderson and May (1981) for general models of invertebrate–pathogen interactions, *i*.*e*., model G, was applied to construct a general *Pochonia*–nematode–root model (GPNR) based on five equations. The model was built using a basic graphic scheme representing the tri-trophic root–nematode and fungus system relationships (**Figure 1A**). Each block in **Figure 1A** represents one of the system components (variable), whereas each arrow is a term in one or more functional equation(s). The constants account for the variable relationships and biology (*i*.*e*., birth rate, egg hatching, mortality, and parasitism). In its discrete, non-derivative form, GPNR simulated, through iterative calculations and for each time *t* (hours) discrete interval, the number of *M. incognita* eggs (variable H), J2 (var. J) and sedentary pre-adult and fecund adult stages (var. A) feeding in a 1-cc rhizosphere microcosm on an amount of host roots (var. R) in the presence of a number of *P. chlamydosporia* propagules (var. V). No egg recovery after fungal infection nor hatching or further re-infection during the fungus infection/transmission process or at its completion were assumed. Due to their rarity, the presence of *M*. *incognita* males was also not considered. The values obtained for each variable at time *t* were used to start the calculation again for the following *t* + 1 interval. For each time interval, the new values were the sum of the calculated values plus those of the preceding time for a number of iterations (4,320 h) equivalent to 180 days. The model begins the calculations for *t* = 1 (first hour) using initial, pre-defined, and estimated values set for each variable as *t* = 0 starting points. In practice, at each iteration, a certain number of eggs hatch or die or a certain amount of J2 penetrate the roots or die and so on. The values obtained for each variable were then used to graphically construct the different curves, overposed on the real data available. The initial variable values and the constants were iteratively tested during running of the program to best fit real data. A screenshot of the model functioning is shown in **Supplementary Figure S1**.

The model equations (1, 5) included two density-dependent factors. The first, the root carrying capacity (*κ*), described how the density of the adult females already present in roots affected a successful J2 establishment and subsequent moulting. The second factor accounted for the effect of root density in the microcosm volume occupied (self-density dependency or soil carrying capacity, *d*).

GPNR used 16 constants describing biological functions including egg release, hatching, moulting, and mortality (see **Table 1** for the definitions of variables and constants, dimensions, and values applied). For modeling, the initial variable and constant values were estimated and assayed as deduced by the literature on fungus or RKN biology, or were directly observed. Hourly nematode moulting or developmental rates throughout the different life stages were inferred from lifetimes obtained through inverse mortality functions on a 24-h basis. The egg production constant was estimated using the number of eggs produced by a fecund female as an egg mass (Ferris et al., 1978; Carlson and Rothfus, 1978; Jeger et al., 1993; Tzortzakakis and Trudgill, 1996), using a total number of up to 1,000 eggs per mass.

**Table 1:** Variables (capital letter), constants, and models used for modeling the interactions between the biocontrol fungus *Pochonia chlamydosporia* and the root-knot nematode *Meloidogyne incognita* in a rhizosphere microcosm.*

Symbol	Value^a^	Model	Biological stage or process described	Dimension
A	0.1	GPNR	Sedentary nematode stages (pre-adults and fertile females)	cc soil^-1^
Ad	0	GPNRd	Sedentary fertile nematode females	cc soil^-1^
F	0	GPNRd	Sedentary pre-adult nematodes	cc soil^-1^
H	5	GPNR	Eggs of *Meloidogyne incognita* (total number)	cc soil^-1^
J	0.1	GPNR, GPNRd	*M. incognita* juveniles (J2 stage)	cc soil^-1^
R	0.25	GPNR, GPNRd	Roots	mg · cc soil^-1^
S	0	GPNRd	Non-parasitic fungus propagules	cc soil^-1^
V	5 · 10^3^	GPNR	Propagules of *P. chlamydosporia*	cc soil^-1^
Vd	5 · 10^3^	GPNRd	Infective fungus propagules	cc soil^-1^
X	5	GPNRd	Healthy nematode eggs	cc soil^-1^
Y	0	GPNRd	Infected nematode eggs	cc soil^-1^
*a*	0.33	GPNR	Percent of fertile, sedentary nematodes	%
*α*	1.75	GPNR, GPNRd	Individual egg production rate	eggs · adult^-1^ · cc^-1^ · mg root^-11^
*b*	2.5 · 10^-3^	GPNR, GPNRd	Egg natural mortality rate	%
*β*	8.75 · 10^-6^	GPNR	Microcosm volume available for conversion from egg to fungus per propagule	cc · propagule^-1^
*β t*	1.75 · 10^-5^	GPNRd	Microcosm volume available for transmission to eggs per propagule	cc · propagule^-1^
*bj*	4 · 10^-3^	GPNR, GPNRd	J2 natural mortality rate	%
*d*	50	GPNR, GPNRd	Root density-dependent factor (soil carrying capacity)	mg root · cc^-1^
*δ*	1 · 10^-4^	GPNRd	Volume of growing root available for feeding	cc · mg root^-1^
*f*	1.4 · 10^-3^	GPNRd	Soil volume available per feeding nematode	cc soil · nematode^-1^
*φ*	1.6 · 10^-1^	GPNRd	Egg to propagule conversion rate	%
*g*	2 · 10^-2^	GPNR, GPNRd	Moulting rate (J2 to sedentary stage)	%
*h*	2.5 · 10^-3^	GPNRd	Egg hatching rate	%
*k*	1.75 · 10^-3^	GPNR	Sedentary stage mortality rate	%
*kt*	2.75 · 10^-3^	GPNRd	Sedentary immature stage mortality rate	%
*ka*	2.8 · 10^-3^	GPNRd	Adult female natural mortality	%
*κ*	500	GPNR, GPNRd	Sedentary stage density-dependent factor (root carrying capacity for nematodes)	nematodes · mg root^-1^
*υ*	6.8 · 10^-3^	GPNR, GPNRd	Root growth rate	%
*p*	60	GPNR	Average number of fungus propagules produced per egg	propagules · egg^-1^
*q*	2.75 · 10^-4^	GPNR, GPNRd	Egg quiescence rate	%
*r*	8 · 10^-4^	GPNR, GPNRd	Root natural mortality rate	%
*ρ*	4.25 · 10^-4^	GPNRd	Microcosm volume available for fungus transmission from infected eggs	cc · egg^-1^
*σ*	2.6 · 10^-4^	GPNRd	Rate of fungus non-parasitic growth	%
*s*	2.75 · 10^-2^	GPNRd	Fungus switch rate to non-parasitic	%
*ζ*	0.4	GPNRd	New propagule switch rate to parasitic	%
*τ*	1.65 · 10^-3^	GPNR, GPNRd	Fungus mortality rate	%
*ω*	7.5 · 10^-3^	GPNRd	Fungus switch rate to parasitic	%

*Densities and constants time scale = hour^-1^.

a Initial variable values and constants.

The constant *g*, accounting for J2 moulting to a pre-feeding stage in root that becomes sedentary, was estimated considering the short time spent in soil by J2 for root location and that root penetration may take less than 24 h, although in soil it can last around a few days. Similarly, the average J2 lifetime in soil in the absence of root penetration (equivalent to bj-1) was inferred as lasting around 10–16 days, as shown by observed or reported ranges of *M. incognita* J2 survival in different *in vitro* or field assays (Starr and Jeger, 1985; Goodell and Ferris, 1989; Ciancio, 1995).

The fungus effect was simulated by testing different initial densities, with growth as a function of the number of eggs. Since *P. chlamydosporia* includes cells, hyphae of different lengths, or spores, its density was generically assumed as a function of propagules, with no distinction among hyphal sectors, cells, conidia, or chlamydospores, also considering that all forms may directly or indirectly start an infection process. In its differential form, GPNR was as follows:


$$\{\begin{array}{ll} \text{dh}/\text{dt}=\text{αaR}-\left(\text{h}+\text{b}+\text{q}\right)H-\text{βH V} & 1)\\ \text{dJ}/\text{dt}=\left(\text{h}+\text{q}\right)\text{H}-\left(\text{bj}+\text{q}\right)\text{J} & 2)\\ \text{dA}/\text{dt}=\text{gJ}\left[1-\left(\text{A}/\text{κR}\right)\right]-\text{k A} & 3)\\ \text{dR}/\text{dt}=\text{υR}\left[1-\left(\text{R}/\text{d}\right)-\text{R}\left(\text{f A}-\text{r}\right)\right] & 4)\\ \text{dV}/\text{dt}=\beta \text{pHV}-\text{τV} & 5) \end{array}$$

### Detailed Model (GPNRd)

Since GPNR cannot provide data on *P. chlamydosporia* prevalence, as the fungus density change in (5) is a function of the egg and fungus previous density through a conversion rate to propagules, the model GPNRd (**Figure 1B**) was developed to describe, in its discrete form, the tri-trophic system with more details.

GPNRd was an expansion of GPNR and also considered the behavior of *P. chlamydosporia* in soil and rhizosphere, in which the fungus can either act as a saprotroph or as an endophyte (Lopez-Llorca et al., 2008). GPNRd included switch rates between non-parasitic (var. S) propagules, including saprotrophic and endophytic stages, and parasitic (var. Vd) propagules. Nematode eggs were assigned to two classes, namely healthy (var. X) or infected by the fungus (var. Y), thus allowing a measure of prevalence. GPNRd also included the different *M. incognita* stages, starting from fecund females (var. Ad) producing healthy eggs (var. X) that hatch as J2 (var. J). The latter migrate inside roots (var. R) and moult to become sedentary immature stages (var. F, including all feeding pre-adult stages) feeding on roots. These stages subsequently become adults (var. Ad) that release eggs to complete the nematode life cycle. The graphic representation of GPNRd is shown in **Figure 1B**.

Two types of transmission sources for the infection of healthy eggs were considered in GPNRd. The first type of transmission is directly originating from hyphae and appressoria from propagules present in the microcosm (var. V). A second source of infection proceeds from infective eggs (var. Y), from which the emerging parasite can directly contact other close, healthy eggs in the mass. The fungus population was also split in a non-parasitic fraction (var. S), growing in soil at a rate *σ*, and a parasitic one (var. V). The rates of switching between the two behaviors are indicated as *s* and *ω*, respectively (**Figure 1B**). Similarly, a further rate of switching between the two trophic behaviors (*ζ*) was also applied to the newly produced propagules. GPNRd included eight equations and 23 constants (see **Table 1** for definitions, dimensions, and values). In its differential form, GPNRd was as follows:


$$\{\begin{array}{ll} \text{dF}/\text{dt}=\text{g J}\left(1-\text{F}/\text{κR}\right)–\text{kt F}-\text{δF R} & 6)\\ \text{dV}/\text{dt}=\text{pφζY}–\text{V}\left(\text{τ}+\text{s}\right)+\text{ωS} & 7)\\ \text{dR}/\text{dt}=\upsilon \text{R }\left(1-\text{R}/\text{d}\right)–\text{f R}\left(\text{F}+\text{Ad}\right)–\text{r} & 8)\\ \text{dAd}/\text{dt}=\text{gf F R}–\text{ ka Ad} & 9)\\ \text{dJ}/\text{dt}=\left(\text{h}+\text{q}\right)\text{X}–\left(\text{g}+\text{bj}\right)\text{J} & 10)\\ \text{dX}/\text{dt}=\text{αAd R}–\left(\text{b}+\text{h}+\text{q}\right)\text{X}-\text{βt X V}-\rho \text{X Y} & 11)\\ \text{dY/dt}=\beta \text{t XV}+\text{ρXY}-\text{φY} & 12)\\ \text{dS}/\text{dt}=\text{sV}+\text{S}\left(\text{σ}-\text{τ}-\text{ω}\right)+p\text{φ}\left(1-\text{ζ}\right)Y & 13) \end{array}$$

GPNRd was assayed by applying the same constant values in common with GPNR. Newly added constants were estimated to allow a sequence of nematode cycles in time, using as a guideline the number of eggs in a mass and other RKN basic biological parameters, as reported in the literature (Ferris et al., 1978; Jeger et al., 1993).

### Analyses and Simulations

The correspondence of simulated and observed values of J2, fungus propagule numbers, or CFUs or observed prevalence values was used for model validation by comparison with the data observed in the trial (Puertas, 2007). Discrete population dynamics were simulated using MathCad 3.1 in a Windows environment and following the Euler numerical method. The initial fungus numbers used for GPNR and GPNRd simulations corresponded to the inoculum (5 · 10^3^ propagules · cc^-1^ soil) used for the biocontrol assays (Puertas, 2007). For nematodes, different initial egg, J2, and adult female numbers were tested, seeing to it that the *M. incognita* female individuals’ yields were not higher than 1,000 eggs per fecund adult.

## Results

### General Model

**Figure 2:**
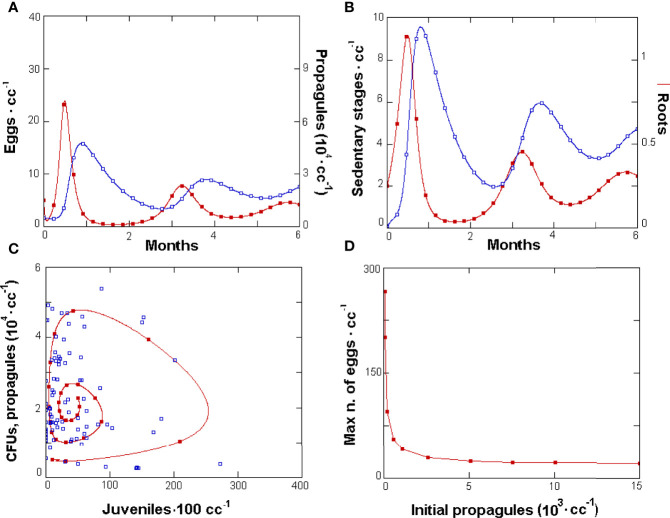
GPNR-simulated dynamics of *Meloidogyne incognita*, *Pochonia chlamydosporia*, and roots. **(A)** Six-month population dynamics of *M. incognita* eggs (filled symbols) and parasitic fungus propagules and **(B)** roots (filled symbols) and nematode sedentary stages. Data were produced with initial *P. chlamydosporia* densities of 5 · 10^3^ propagules · cc soil^-1^. GPNR validation with observed data **(C)**: phases space plot of simulated propagule numbers (filled symbols) resulting from the model cycle, and of observed mean colony-forming units from soil and roots · cc^-1^ *vs*. simulated and observed J2 · 100 cc soil^-1^ (empty squares). The starting values for other variables were as follows: 5 eggs · cc soil^-1^, 0.1 adults · cc soil^-1^, 0.1 J2 · cc soil^-1^, and 0.25 g roots · cc soil^-1^. **(D)** Effect of increasing initial fungus densities on the maximum number of eggs · cc soil^-1^ observed in the cycle.

GPNR allowed the simulation of the tri-trophic relationships and a first representation of the effects of each component considered, yielding a stable structure of interactions as shown by the density changes and the simulated population dynamics data (**Figures 2A,B**). The model yielded stable cyclic relationships, observed on the phases space given by the fungus propagule *vs*. J2 densities, that matched the observed CFUs *vs*. J2 data (**Figure 2C**). The relationship of *M. incognita* with *P. chlamydosporia* was mainly of the density-dependent type, with shifted peaks alternated in time (**Figure 2A**). A similar relationship was also observed for roots and RKN sedentary stages (**Figure 2B**). Recursive, cyclic trends were not regular, and the simulated cycle fitted real data mainly confined to an attractor region where the latter observations aggregated (**Figure 2C**).

Simulations confirmed that the *P. chlamydosporia* optimal initial density was 5 · 10^3^ propagules · cc soil^-1^, a value applied in experimental assay. No further reduction in the maximum number of nematode eggs was observed beyond this threshold (**Figure 2D**). Adherence to real microcosm conditions and nematode biology was confirmed by the number of eggs yielded per egg mass (1/k · α = 10^3^). Similarly, the J2 average lifespan (bj · 24)^-1^ = 10.4 days fitted the nematode survival usually expected in soil, as did the time lapse required to enter roots (g · 24)^-1^ = 2.1 days, the eggs’ lifespan (b · 24)^-1^ = 16.7 days, and the quiescence period, lasting around (q · 24)^-1^ = 151 days. The eggs’ hatching rate, set as equivalent to their mortality, forced the eggs to be hatched or dead at the end of this period. The adults’ lifespan—(k · 24)^-1^ = 23.8 days — fitted the nematode biology. Similarly, the fungus mortality rate allowed an average propagule lifespan lasting (*τ* · 24)^-1^ = 25 days, whereas the average feeder root lifespan was (*r* · 24)^-1^ = 52 days.

### Detailed Model

**Figure 3:**
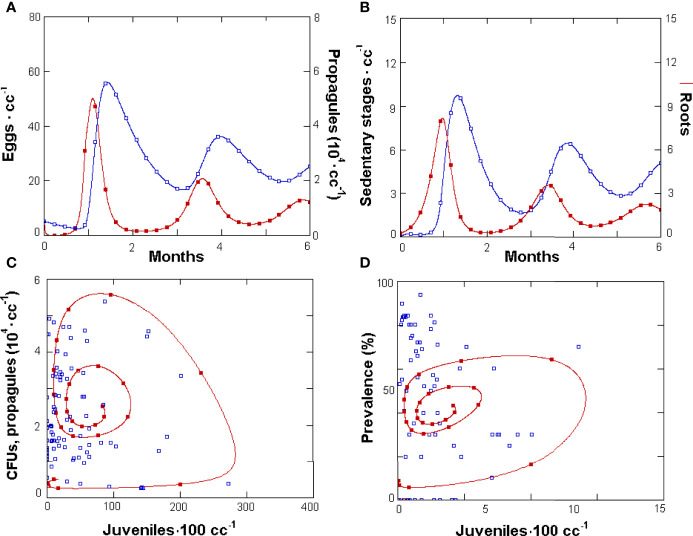
Density changes simulated by GPNRd in a 6-month period for *Meloidogyne incognita* eggs (filled symbols) and *Pochonia chlamydosporia* propagules · cc soil^-1^ **(A)**, and for feeder roots · cc soil^-1^ (filled squares) and nematode sedentary stages **(B)**. Phases space plot **(C)** of GPNRd-simulated propagule numbers (filled symbols) and observed mean soil and roots CFUs · cc^-1^ (empty squares) *vs*. simulated and observed J2 · 100 cc soil^-1^, respectively. **(D)** Phases space plot of GPNRd-simulated (filled symbols) and measured fungus prevalence in eggs (for initial points and constants see **Table 1**).

A more detailed insight on the tri-trophic system was provided by GPNRd, which yielded simulated densities for each nematode life stage. This was achieved by splitting the sedentary stages used in GPNR in two classes: the immature and the adult fecund females, respectively (**Figure 1B**). Similarly, GPNRd yielded data for the two different fungus populations (parasitic or non-parasitic) as well as prevalence values. The model had 13 constants in common with GPNR and showed similar egg and J2 population dynamics, although with higher nematode numbers (**Figure 3A**). Similar results, with higher values, were also shown for RKN sedentary stages and roots (**Figure 3B**), whereas the propagule numbers and CFUs *vs*. J2 cycle showed a wider amplitude (**Figure 3C**).

**Figure 4:**
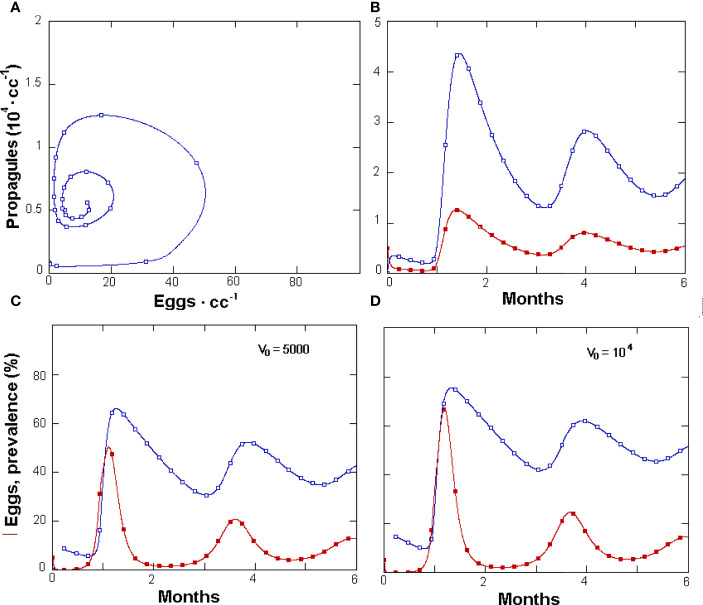
**(A, C)** Phases space relationship between *Pochonia chlamydosporia* parasitic propagules and *Meloidogyne incognita* eggs **(A)** and related density changes in 6 months in a GPNRd-simulated rhizosphere microcosm. **(B)** Six-month population dynamics of parasitic (filled squares) or non-parasitic fungus propagules. Data produced by GNPRd at different initial parasitic propagule densities **(C, D)** (for initial variables and constants see **Table 1**).

The fungus prevalence showed a cyclic relationship with the J2 numbers and approached the observed data: 38.3% ± 15.2 *vs*. 39.4% ± 33.1 (mean ± SD, respectively). For GPNRd, the simulated observations also concentrated towards an attractor or equilibrium region on this phase space, which aggregated the majority of real observations (**Figure 3D**). Similarly, on the eggs *vs*. Fungus phase space, GPNRd showed a cyclic relationship of simulated data, which aggregated in an attractor region (**Figure 4A**). GPNRd also allowed the evaluation of the fungus switch between the parasitic and non-parasitic propagule fractions, with a clear predominance of the latter (**Figure 4B**).

**Figure 5:**
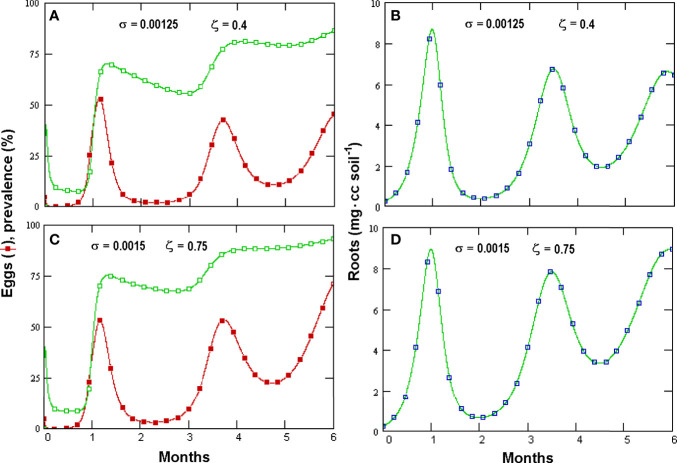
Effects of increasing *Pochonia chlamydosporia* non-parasitic growth rate (*σ*, insets) and propagules switch to parasitism (ζ) on prevalence (empty squares) and *Meloidogyne incognita* egg densities (filled symbols) **(A**, **C)** and root density **(B**, **D)** as simulated by GPNRd (for initial variables see **Table 1**).

**Figure 6:**
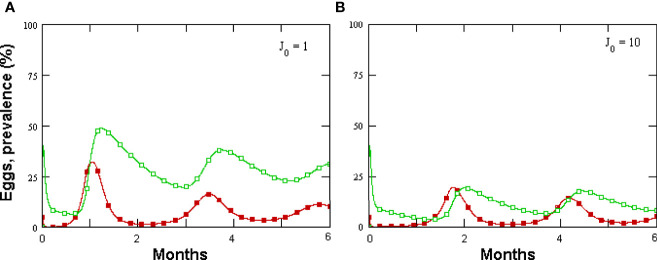
**(A, B)** Effect of varying intial J2 numbers on *Meloidogyne incognita* eggs in 1 cc rhizosphere microcosm (filled symbols) and prevalence, as modeled by GPNRd.

**Figure 7:**
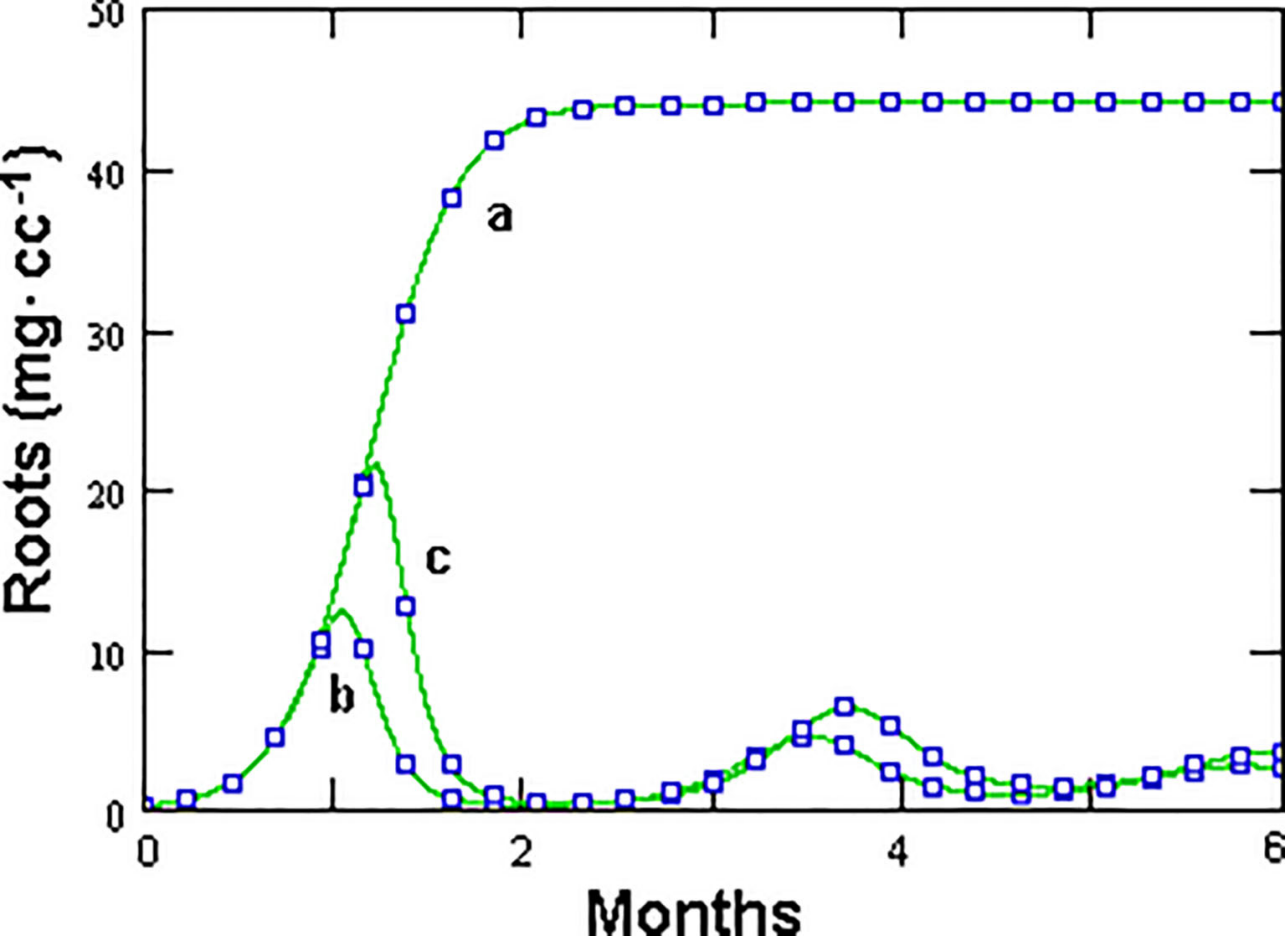
Effect of the initial number of healthy eggs, as unique nematode source, on root growth, as modeled by GPNRd, with *X*_0_ = 0 **(a)**, *X*_0_ = 1 **(b)**, and *X*_0_ = 100 **(c)** eggs in 1 cc rhizosphere microcosm.

Prevalence showed a briefly delayed dependence on egg numbers, with higher egg densities observed when increasing the initial fungal amounts—a condition yielding, however, only a minor increment in prevalence (**Figures 4C, D**). A significant, density-independent effect on prevalence, exerted through the non-parasitic-parasitic switch, was observed when progressively increasing the rate of *P*. *chlamydosporia* non-parasitic growth (**Figures 5A, C**). This effect was balanced by roots since, even at high levels of σ and *ζ*, the nematode population never became extinct (**Figures 5A, C**), being sustained by the increased amounts of roots (**Figures 5B, D**). GPNRd simulations showed that the density of *P. chlamydosporia* affected the RKN numbers in a non-linear way and that varying initial conditions (*i*.*e*., J2 numbers) originated cycles of different amplitudes and/or nematode densities, inducing a shift in the time of highest egg peaks (**Figures 6A, D**). Initial conditions also affected the total amount of roots obtained, as shown by the reduction in root growth observable even at low initial nematode egg numbers (**Figure 7**).

## Discussion

Both models were informative about the components of the tri-trophic system under examination, yielding data for the *M. incognita* population compatible with the host nematode dynamics usually observed in tropical crops (Ciancio and Quénéhervé, 2000; Ehwaeti et al., 2000; Puertas, 2007). Differences in model resolution suggest different applications, with GPNR appearing to be simpler and suitable to interpret observations from real assays due to the lower number of constants and variables involved. GPNRd appears helpful when investigating *P. chlamydosporia* behavior and the feedback effects induced by the changes, *i*.*e*., of root amounts or increased prevalence. In both cases, however, the complexity of the real system was matched only partially since, *i*.*e*., the chlamydospore stage of *P. chlamydosporia* and its endophytic behavior were not considered. Other factors excluded were local synergy or competition effects. Due to complexity and stochasticity or real field populations, GPNR and GPNRd predictability may also hold only for a short (few weeks) period. However, both models were useful to identify and test key system components and mechanisms as well as to show the occurrence of attractors in the rhizosphere microcosm.

Dependence on initial conditions is known to affect nematode reproduction rates (Seinhorst, 1967), suggesting that in real systems (*i*.*e*., field crops) even small perturbations at the beginning of a cycle may have a significant impact on the overall system evolution. This effect may have practical implications, *i*.*e*., in nematode management, but it will require, for application, a previous monitoring of nematode stages and fungus densities as well as the evaluation of other environmental variables influencing their evolution.

For validation, GPNRd showed higher stability and yielded data on healthy or infected eggs as balanced by the *P. chlamydosporia* parasitic and non-parasitic fractions. Prevalence levels around 60% were enough to avoid either root or nematode extinctions, fitting the experimental data observed in the long-term assay. The attractors showed on the phase spaces by both models (**Figures 2C**, **3C, D**, **4A**) provide a possible framework to interpret nematode suppressivity as observed in susceptible crops in case, *i*.*e*., the attractor can keep, during a crop cycle, the nematode density values below any damage threshold. However, both models do not consider the presence of *P. chlamydosporia* in soil or its survival in the absence of roots or nematodes. Although the fungus grows in close contact with roots infested by *M. incognita*, relying on egg parasitism or on nutrients released in the rhizosphere (Kerry, 2001), it can also survive as a saprotroph on various substrates like chitin, cellulose, or other invertebrates in the absence of roots. GPNRd simulations, however, showed that both fractions of the fungus decreased in the absence of nematodes (data not shown), suggesting that, at least in the simplified model systems, parasitism is a necessary survival strategy for the fungus. This effect is in accordance with the general concept that nematophagous fungi exploit nematode parasitism as an additional food source rather than as a unique trophic target.

The constants applied yielded a realistic sub-optimal RKN life cycle lasting less than 2 months. In optimal conditions (28°C), *M. incognita* lifetime lasts around 4 weeks, dropping at 9 weeks with sub-optimal temperatures (Ploeg and Maris, 1999). For simulations, the egg death rate *b* matched the rate reported for the eggs of *M. arenaria*, a close species, for which 36% mortality was observed *in vitro* after 1 week at 21°C, corresponding to a rate of 2.3 · 10 ^-3^ · hour^-1^ (Ferris et al., 1978). The rate observed, however, referred to the period after egg collection and not to the true egg age (days after release) and significantly decreased during the following weeks (Ferris et al., 1978), suggesting that this process may be a function of several variables, including other mortality or environmental factors not considered in the models.

An exception to the determination of constants through lifetimes was the hatching rate *h*, accounting for the individual hatching probability, whose inverse value accounts for more than 2 weeks. The egg biology, including delayed hatching of eggs in diapause, also required the introduction of constant *q*, incorporating a minor fraction of eggs falling in diapause and hatching with a time delay of around 150 days (De Guiran, 1980; Starr and Jeger, 1985; Huang and Pereira, 1994).

Constants *β* and *βt* account for the microcosm volume available for parasitism transmission for any propagule (GPNR) or for an infective one (GPNRd), respectively. The transmission constant *ρ* in GPNRd accounts for the eggs infected per egg already parasitized, whereas *φ* provides the number of eggs converted to fungus per propagule in the microcosm. These constants describe direct interactions between fungus and eggs, both approximating more complex functions, and their definition will require further experimental assays.

The density dependence (*d*) factor controls the total amount of roots which can be reached in the absence of nematode parasitism (microcosm carrying capacity). In this case, an estimate of 50 mg of roots · cc^-1^ was used, but this value may largely vary depending on the host plant and growth conditions experimented. It is worth noting that both models, although allowing a realistic root growth, did not lend space to intermediate situations, *i*.*e*., a significant growth in the presence of a RKN population controlled only by the fungus. Simulations with GPNRd using different initial values of healthy eggs only showed that the fungus alone could not warrant significant root growth even in the presence of a very low initial nematode inoculum (**Figure 7**). This aspect derives from the inverse relationship between initial nematode density and growth rate and requires further investigations since a wide range of intermediate situations are usually observed in RKN-infested roots.

**Figure 8:**
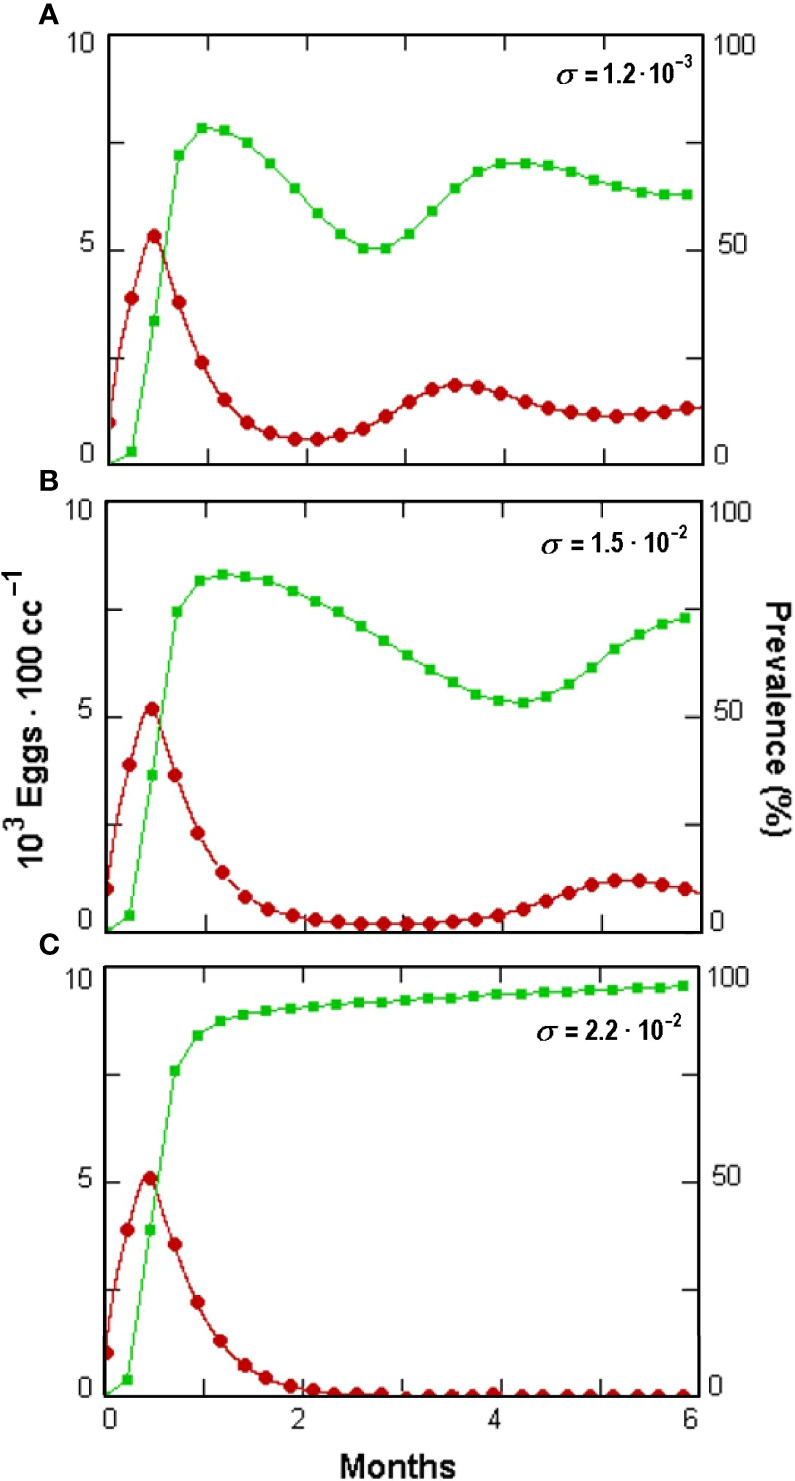
Effect **(A–C)** of increasing *Pochonia chlamydosporia* non-parasitic growth rate (*σ*, insets) on prevalence (green squares) and *Meloidogyne incognita* egg densities (red dots) as simulated by GPNRd, showing host extinction at highest *σ* (see **Table 1** for the initial values of the variable).

The non-parasitic growth of *P. chlamydosporia* also affected biocontrol through the fungus switch to parasitism (**Figures 8A–C**). This mechanism offers a novel perspective for this fungus since its poor non-parasitic performance was considered to reduce its competition in the rhizosphere (Bailey et al., 2008). It holds, however, only when *ω* > *s*, a condition not available in GPNRd with the constants used due to the insurgence of chaotic oscillations. These components are worth further investigations since the parasitic propagules increase in a host-independent way, as they are recruited from the non-parasitic fungus population at the rate of *ω*.

The non-parasitic growth rate *σ* and the switch *ζ* of newly produced propagules also affected the simulated dynamics through a density-independent regulation (**Figures 5A, C**). This property may have practical implications in real systems, confirming that the non-parasitic fungal behavior is a key biological process (De Castro and Bolker, 2005). Whether and to what extent this switch occurs in the rhizosphere can be determined only by discriminating live saprotrophs or endophytes from parasitic propagules through the detection of, *i*.*e*., specific parasitism-related RNAs or metabolic products (Ward et al., 2012).

## Conclusions

The relationships linking biocontrol agents and their nematode targets are complex, and their description requires many variables related to the biology of all the organisms involved. Although GPNR and GPNRd predictability holds only for a few weeks and depends largely on initial conditions, the simulations resulted informative about the tri-trophic root–*M. incognita*–*Pochonia* system functioning. GPNR was simpler and suitable to interpret data from real assays due to its lower number of constants and variables. GPNRd was more detailed, showing a higher stability in yielding simulated data about the number of healthy or infected eggs as regulated by the fungus parasitic and non-parasitic fractions. The density dependence of parasitism, the transmission coefficients regulating parasitism likelihood, and the self-density dependence factors controlling all organisms involved appeared as factors of major importance. The non-parasitic growth of *P. chlamydosporia* also affected nematode regulation through the fungus propagule switch from a non-parasitic behavior to parasitism. This factor has practical effects in real systems, confirming that the non-parasitic fungal behavior is a key biological process affecting biocontrol efficacy through a further density-independent host regulation mechanism.

## Data Availability Statement

The original contributions presented in the study are included in the article/**Supplementary Material**. Further inquiries can be directed to the corresponding author.

## Author Contributions

LHD, IMC and AP designed the nematode experiments. AP, LHD and YCD performed the experiments and the data collection. AC constructed the models. AC and IMC tested and applied the models to experiment data. AC, IMC and AP drafted the manuscript. AC and IMC wrote the final version of the manuscript. All authors edited and approved the final manuscript.

## Funding

This research was partially funded by EU Project ICA4-CT-2002-10044, MicoSpa: “Microbial Pest Control for Sustainable Peri-urban/Urban Agriculture in Latin America (Mexico and Cuba)”. AC also acknowledges partial funding by MIPAF (Project BIOMED) and CNR (Project CISIA).

## Conflict of Interest

Author LH-D is employed by the company Laboratorios Biologicos Torres.

The remaining authors declare that the research was conducted in the absence of any commercial or financial relationships that could be construed as a potential conflict of interest.

## Publisher’s Note

All claims expressed in this article are solely those of the authors and do not necessarily represent those of their affiliated organizations, or those of the publisher, the editors and the reviewers. Any product that may be evaluated in this article, or claim that may be made by its manufacturer, is not guaranteed or endorsed by the publisher.
