# Supplementary material for: Modeling Root-Knot Nematode Regulation by the Biocontrol Fungus Pochonia chlamydosporia
Source: Front Fungal Biol. 2022 Jul 26;3:900974. doi: 10.3389/ffunb.2022.900974 (PMC10512345; doi:10.3389/ffunb.2022.900974)

Supplementary Figure 1. A screenshot of the Mathcad GPNR model file, showing the iterative calculations, initial variable settings, the constants, the system of equations and the iterative graphic data displays. For variables and constants descriptions see Figure 1 and Table 1.

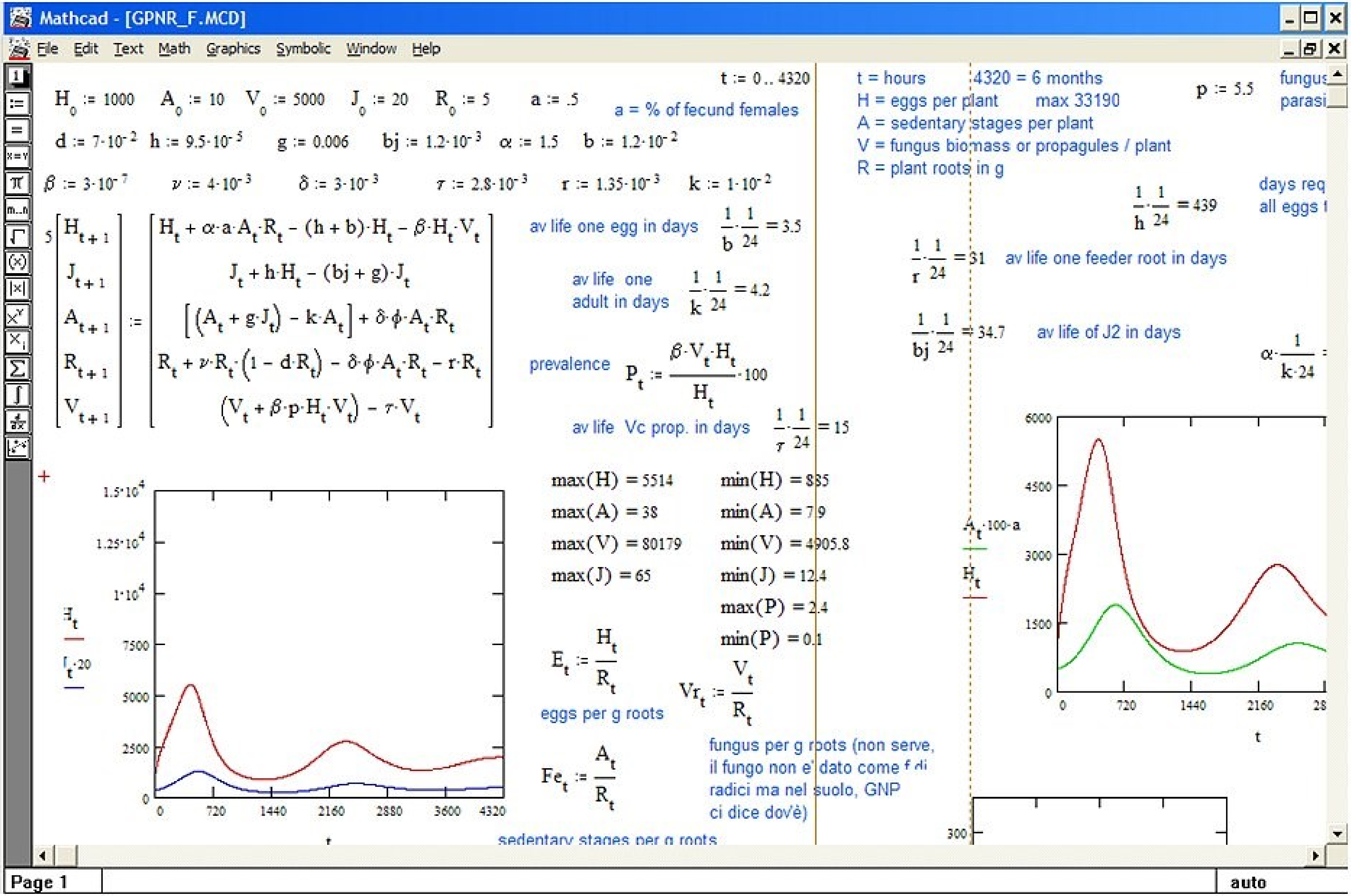

Supplement: Supplementary file 2 [file Image_1.pdf]
